# Altered resting-state functional connectivity in a thalamo-cortico-cerebellar network in patients with schizophrenia

**DOI:** 10.1038/s41598-024-78297-3

**Published:** 2024-11-01

**Authors:** Caroline Garcia Forlim, Leonie Klock, Jürgen Gallinat, Simone Kühn

**Affiliations:** 1 Clinic and Policlinic for Psychiatry and Psychotherapy, University Medical Center Hamburg-Eppendorf, Martinistraße, 52, W37, EG, Room 107/109, 20246 Hamburg, Germany; 2 Center for Environmental Neuroscience, Max Planck Institute for Human Development, Berlin, Germany; 3 Berlin School of Mind and Brain, Humboldt-Universität zu Berlin, Berlin, Germany

**Keywords:** Neuroscience, Schizophrenia

## Abstract

The diagnosis of schizophrenia is associated with a complex psychopathology related to disrupted brain circuitry causing a failure in coordinating information across brain sites with no consensus regarding the mechanisms. Although schizophrenia is well-studied, the great majority of studies investigated pre-selected ROIs or Seed-based connectivity. Whole brain ROI-wise studies that consider all ROIs available simultaneously are lacking. This technique helps understand large- and local-scale dynamics of information exchange across the whole brain. We investigated ROI-wise whole brain networks in 35 participants diagnosed with schizophrenia and 41 control participants. To unveil dysfunctions in brain subnetworks and to characterize network topology, we applied a statistical tool specially developed for network comparison called network-based statistic and graph theory. We observed a hyperconnected thalamo-cortico-cerebellar subnetwork in participants with schizophrenia; nodal analysis revealed higher number of thalamic connections. Our results suggest disruptions at the local level of the subnetwork rather than globally spread across the brain and driven by hyperconnectivity. Importantly, this subnetwork emerged from an exploratory analysis directly comparing ROI-wise whole brain network. This fact makes it an important contribution to the field as additional evidence, demonstrating the high reliability of malfunction in the local thalamo-cortico-cerebellar network.

**Supplementary Information:**

The online version contains supplementary material available at 10.1038/s41598-024-78297-3.

## Introduction

The diagnosis of Schizophrenia describes a mental disorder that includes alterations in cognitive processes, perception, affect, and the sense of self^1^. Within a lifetime, around 7 out of 1000 individuals are diagnosed with schizophrenia emphasizing the importance of this disorder to public health^2,3^.

From a neurophysiological perspective, the research focus of schizophrenia has moved from investigating impaired localized brain regions to studying abnormal interactions between brain regions^4,5^, known as connectivity, giving rise to models of altered neural connectivity that claim to explain the heterogeneity of symptoms distinctive for schizophrenia. Prominent examples of this new paradigm taking interactions between brain regions into consideration, are the cognitive dysmetria and disconnection hypothesis^4–6^). Thanks to neuroimaging techniques like functional magnetic resonance imaging (fMRI), a growing body of studies reported abnormal functional connectivity (FC) within the last years, where aberrant brain circuitry causes a failure in coordinating information across multiple brain sites^7–11^.

Although schizophrenia is, at first sight, a well-studied disorder, the great majority of studies investigated pre-selected regions of interest (ROIs) or Seed-based FC, known as hypothesis-based studies. For this reason, whole brain ROI-wise studies where all ROIs available are taken simultaneously into account are lacking. However, these studies, are of great importance because it is the only technique that allows to understand the complete picture of the brain unveiling whether and how large- and local-scale dynamics of information exchange across the whole brain differs in patients with schizophrenia from healthy controls. To the best of our knowledge, there is a limited amount of resting-state fMRI studies (< 10) to this day that included all ROIs available in the brain, performing effectively what is called whole brain functional network comparison.

The lack of studies comparing whole brain FC between groups is due, in great part, to the complexity of dealing with large scale networks. In resting-state fMRI, functional networks are often constructed based on brain areas obtained using ROIs from anatomical templates and the connectivity is inferred using correlations between brain areas. A network is comprised of nodes (ROIs) that are connected via links (functional correlations) between nodes. In order to find potential differences between groups of individuals, the network comparison is usually conducted pairwise between all links^12,13^. This pairwise comparison leads to a large number of comparisons and therefore, a multiple comparison problem, for example, whole brain analysis of 100 ROIs give rise to network of 4.950 links. To overcome this issue and yet controlling for family-wise error, a statistical tool called network-based statistics (NBS) has been developed^12^ and used in the present analysis.

NBS has been applied to patients with schizophrenia in studies using multiple brain imaging techniques: EEG, MRI, task-fMRI and resting-state fMRI. Focusing only on resting-state fMRI studies, where links were calculated using Pearson’s correlation to account for the connectivity, in whole brain networks built by using small regions of interest (ROIs) derived from AAL atlas as nodes, revealed impaired subnetworks comprising occipital, temporal and orbitofrontal regions^14^. In those built with classic AAL partition, impairment was shown in 3 subnetworks encompassing parieto-temporal connections, occipito-parietal, occipito-temporal, and parietal–temporal interactions and fronto-temporal, and fronto-striatal regions^15^. A voxel-wise approach revealed impaired fronto-parietal and occipital-parietal subnetworks^16^. A study with recovered patients from schizophrenia spectrum disorders or other psychotic disorders in comparison with healthy controls showed impaired in cingulo-opercular network and occipital gyrus^17^). Considering treatment-resistant patients, impairment was found in a subnetwork comprising temporal, frontal and occipital cortices^18^. In a clinical population with high risk for psychosis in comparison to controls showed no differences in resting-state connectivity in turn, when using multi-paradigm fMRI data and principal component analysis in NBS, they found an impaired cerebello-thalamo-cortical subnetwork^19^. Additional studies have used wavelet to infer the FC: the first found an impaired subnetwork comprising fronto-temporal and occipito-temporal areas^12^ and the second one investigated treatment-resistant schizophrenia observing impairment between cerebellar and parietal regions to the frontal cortex^20^. A third study showed mainly increased connectivity in fronto-temporal and fronto-insula cingulate areas in patients with schizophrenia and non-clinical individuals with hallucinations^21^. We would like to bring your attention to the fact that, in the previously mentioned studies, the choice of the nodes was heterogeneous regarding the size of the brain regions as well as the number of brain regions. For instance, none of the studies comparing ROI-wise whole brain networks using NBS have included cerebellar regions except for the one that investigated treatment-resistant patients^18,20^. Nonetheless, the low number of studies is understandable and even expected because including cerebellar regions poses an additional challenge in the analysis process since image acquisition protocols typically prioritize frontal areas and oftentimes cut off cerebellar regions. Thus, further verification steps are needed to ensure quality control. While the choice of the nodes in network analysis is an individual decision and all studies mentioned above have provided important contribution to the field, it is important to point out that, by not including cerebellar regions, valuable information on their role in the whole brain connectivity could have potentially been missed.

Another widely used method to investigate large-scale networks is called graph analysis where FC maps can be characterized by means of network topology. Graph analysis has been applied to structural and functional brain networks. Narrowing it down to the interest of this paper, we focused the reviewed literature on studies using resting-state fMRI. The size of the functional networks as well as the choice of graph measures are heterogeneous which, not surprisingly, have led to disparate results: no significant differences between patients with schizophrenia and healthy controls^22,23^, significant differences in global but not local efficiency^24^, differences in small-worldness, degree, strength and cluster coefficient, but neither multiple comparison correction nor nonparametric statistics were considered^25^ as well as differences in strength and small-worldness^26^. The study of community structures in the brain revealed similar network community structures between healthy controls and patients at the group level and small alterations at the individual level^27^. When considering only subjects with auditory hallucination in comparison with healthy controls, significant differences were found in strength and betweenness^28^. Looking at treatment-resistant schizophrenia, reduced strength and global efficiency and increased local efficiency^18^ as well as unmedicated patients with schizophrenia showed reduced global efficiency and increased clustering coefficient^29^ .

Previously we have analysed connectivity changes within the default mode network^30^ and in the present study, we aimed to investigate, in a data-driven approach, potential differences in information processing in large-scale whole brain resting-state FC, including cerebellar regions, in a group of patients diagnosed with schizophrenia and a group of matched healthy individuals. Our exploratory analysis focused on appropriate large scale network tools to compare ROI-wise whole brain network between groups namely, NBS for a direct comparison of connectivity pathways to unveil FC disruptions and graph analysis to study the topological characteristic of whole brain networks. These tools are complimentary and together allow for the understating of potential differences in the local and global connectivity in patients.

## Materials and methods

### Participants

Forty-one healthy individuals and thirty-five individuals who met the criteria for a diagnosis of schizophrenia following the International Classification for Diseases and Related Health Problems (ICD-10) were included in the reported analysis. Patients were recruited at St. Hedwig Hospital of the Charité-Universitätsmedizin Berlin (Germany). The severity of symptoms was rated by a trained clinician with the Scale for Assessment of Negative Symptoms (SANS)^31^ and the Scale for Assessment of Positive Symptoms (SAPS)^32^. For more details on the assessment of psychopathology please refer to^30^. For details of patients’ psychopathology see Table 1 and Table S1 in the supplementary material for self-reported substance use. Healthy individuals who did not fulfil the criteria for any mental disorder and were not in current or past psychotherapy of an ongoing mental health-related problem were recruited via online advertisement and flyers. None of the participants met the MRI exclusion criteria of claustrophobia, neurological disorders and metallic implants. Healthy individuals were recruited who matched the sample of patients with regard to age, sex, handedness and level of education (Table 1). Handedness was assessed with the Edinburgh Handedness Inventory (*n* = 75), level of cognitive functioning was acquired with the Brief Assessment of Cognition in Schizophrenia^33^ (*n* = 65) and verbal intelligence with a German Vocabulary Test (*n* = 72)^34^. Groups were fully matched for age, sex, handedness and level of education (Table 1). All procedures were approved by the ethics committee of the Charité-Universitätsmedizin Berlin. All subjects gave written informed consent in accordance with the Declaration of Helsinki.

### Data acquisition

Images were collected on a Siemens Tim Trio 3T scanner (Erlangen, Germany) with a 12-channel head coil. Structural images were obtained using a T1-weighted magnetization prepared gradient-echo sequence (MPRAGE) based on the ADNI protocol (TR = 2500ms; TE = 4.77ms; TI = 1100ms, acquisition matrix = 256 × 256 × 176; flip angle = 7˚; 1 × 1 ×  1mm^3^ voxel size). Whole brain functional resting state images during 5 min were collected using a T2*-weighted EPI sequence sensitive to BOLD contrast (TR = 2000 ms, TE = 30ms, image matrix = 64 × 64, FOV = 216 mm, flip angle = 80º, slice thickness = 3.0 mm, distance factor = 20%, voxel size 3 × 3 × 3mm^3^, 36 axial slices). Before data acquisition, participants were in the scanner for about 10 min during which a localizer and the anatomical images were acquired so that subjects could get used to the noise. During data acquisition participants were asked to close their eyes and relax.

### Data preprocessing

The first 5 images were discarded due to steady-state longitudinal magnetization. Firstly, slice timing was applied and then the data was realigned. Structural individual T1 images were coregistered to functional images followed by segmentation into grey matter, white matter, and cerebrospinal fluid. Data was then spatially normalized to the MNI template. To improve signal-to-noise ratio, a spatial smoothing with a 6-mm FWHM was done. Motion parameters in the rp_* file (x-, y-, z-directions, pitch, roll and raw) and signals from white matter and cerebrospinal fluid were regressed, followed by data filtering (0.01–1 Hz). Finally, the data was detrended. In addition, to control for motion, the voxel-specific mean framewise displacement (FD;^35^) was calculated. FD values were below the default threshold of 0.5 for control and patient group (0.15 ± 0.02 and 0.17 ± 0.02, t-test *p* = 0.42). All steps were done using SPM12 except filtering, which was applied using REST-toolbox^36^.

### Network analyses

We used complimentary network tools appropriate for the study of large-scale brain networks. To explore potential differences in information processing, we analysed the ROI-wise connectivity between all ROIs available across the whole brain using NBS. Additionally, we characterized topological properties of whole brain networks using graph analysis. For that, first the whole brain networks were built, which we explain in the following subsection.

**Table 1:** Demographics.

	Healthy Participants Mean (SD)	Schizophrenia Patients Mean (SD)	Statistics T (DF)	*P* value
Sociodemographic characteristics				
Age (years)	35.2 *(11.0)*	35.3 *(10.8)*	-0.059 *(74)*	0.953
Gender	24 male/17 female	21 male/14 female		
Edinburg handedness inventory^a^	79.4 *(38.5)*	75.56 *(52.6)*	0.331 *(59)*	0.742
Education (years)	14.1 *(2.9)*	13.1 *(3.8)*	1.345 *(74)*	0.183
BACS^a^	270.4 *(37.0)*	234.1 *(28.4)*	4.427 *(63)*	< 0.001
Verbal intelligence (IQ)^a^	100.5 *(10.6)*	94.4 *(12.8)*	2.204 *(70)*	0.031
Psychopathology				
Becks depression inventory (BDI)^a^	4.6 *(4.0)*	12.1 *(7.9)*	-5.246 *(72)*	< 0.001
Illness duration (years)		9.4 *(8.8)*		
Illness onset (age in years)		25.6 *(8.9)*		
Chlorpromazine-equivalent (mg)		317.1 *(221.6)*		
SANS composite score^a^		20.2 *(12.0)*		
SAPS composite score^a^		15.4 *(14.9)*		
Extrapyramidal syndrome		None = 30, light = 3		
Fagerström test for nicotine dependence (FTND)^a^	1.2 *(1.7)*	3.8 *(3.1)*	-4.702 *(72)*	< 0.001

*SD* standard deviation, *BACS* Brief Assessment of Cognition in Schizophrenia, *SAPS* Scale for Assessment of Positive Symptoms, *SANS* Scale for Assessment of Negative Symptoms.

^a^Sum score of items reported.

#### Building ROI-wise whole brain functional networks

**Fig. 1:**
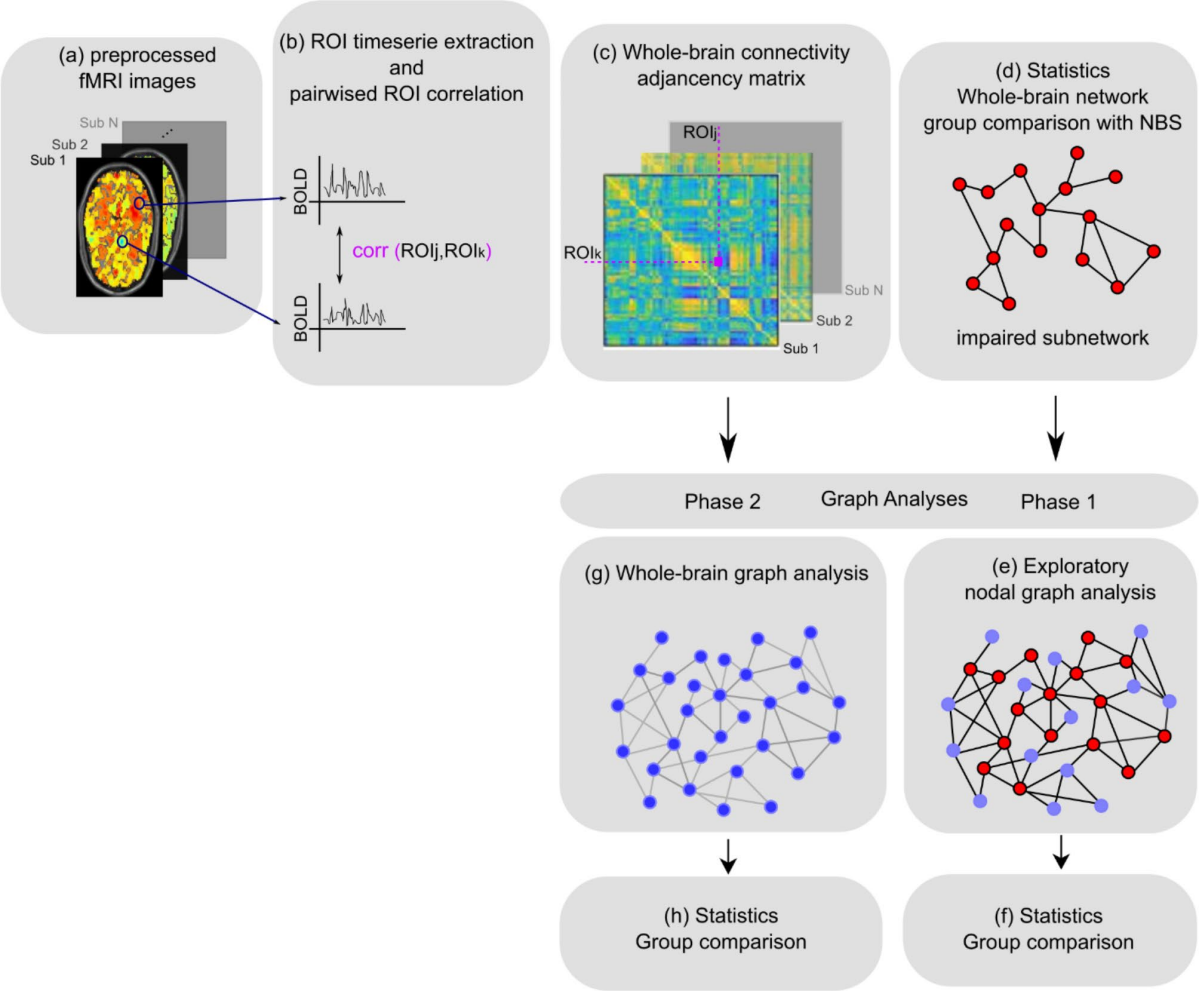
Analysis workflow. Resting state fMRI images were preprocessed (**a**) then the average timeseries of each ROI (AAL atlas) was extracted. (**b**) Next, a pair-wise correlation of all timeseries using Pearson’s correlation coefficient formed (**c**) the adjacency matrix. (**d**) Statistical analysis was performed using Network-based statistics to extract the impaired subnetwork between groups. (**e**) Graph analysis Phase 1—nodal graph analysis using nodes retrieved in (**d**). (**f**) Non parametric t-test of nodal graph measures calculated in (**e**). (**g**) Graph analysis Phase 2—whole brain graph analysis. (**h**) Non parametric t-test of graph measures calculated in (**g**).

To build functional networks (Fig. 1), the nodes were created based on the AAL atlas^37^. Due to the fact that in the analysis process since image acquisition protocols typically prioritize frontal areas and oftentimes cut off cerebellar regions, ROIs in the cerebellum may be cut. To ensure the quality of the networks, ROIs of which the time series could not be estimated for all participants were excluded. The resulting network comprised of 106 nodes. The node-averaged time series of BOLD signal were extracted using the REST-toolbox^36^. The links between all 106 nodes were calculated using Person’s correlation coefficients (Fig. 1a–c). Afterwards, to avoid including weak signals as well as not statistically significant links, a statistical threshold was applied assuring that only significant (*p* < 0.05) connections were taken to the next analysis steps.

#### Graph analysis

The topological properties were calculated using graph measures in the Brain Connectivity toolbox^38^. The following measures were considered: degree, betweenness, characteristic path length, efficiency, diameter and cluster coefficient. For the description of the measure please refer to the supplementary material.

Graph analysis was conducted in two phases: In phase 1 (Fig. 1e,f), exploratory nodal analysis using nodes retrieved from the subnetwork were obtained after statistical comparison to address the lack of specificity of the NBS. Due to the cluster-wise nature of the statistical tool used to compare the whole brain networks, the NBS, no inference can be made about single nodes and links from the impaired local subnetwork, resulting in a lack of specificity. Therefore, in order to explore the global role of individual nodes belonging to the subnetwork revealed by NBS, we calculated their nodal topological characteristics in the whole brain network (Fig. 1e,f): degree, betweenneess and cluster coefficient. In phase 2 (Fig. 1g,h), graph analysis was applied to the whole brain network to characterize and compare the topology of the networks between groups.

Graph measures are typically applied to thresholded brain networks. Nevertheless, there is no consensus about the method, therefore it should be based on an educated guess^39^. We used absolute thresholding ranging from 0 to 0.8 and weighted links as we believe that the strength of the connections plays a role in schizophrenia.

### Statistics

#### ROI-wise whole brain FC comparison

In this study, a two-sample t-test in NBS was applied to FC networks (Fig. 1d) between groups. NBS is a nonparametric method developed to compare large scale networks. NBS uses a cluster-based threshold of statistical parametric maps^12^. In contrast to other methods, instead of performing statistics in voxel clusters, NBS uses connected graph components, namely, by brain areas successively connected by links. First, a set of suprathreshold links is constructed from statistical tests of each link that surpasses a threshold within the whole brain network. Then, the connected graph components are calculated from this set of suprathreshold links using a breadth-first search algorithm and the size of the connected components is stored. The *p*-value of an observed connected component of a certain size is calculated, using permutation testing. To estimate the null distribution, N random permutations are done. The disadvantage of the method is the lack of specificity, as statistics are performed on connected graph components and thus no inferences can be made about individual links and nodes. For further details please refer to^12^.

In the NBS toolbox we set *N* = 10,000 permutations to estimate the null distribution. The choice of the suprathreshold is an intrinsic and arbitrary step in NBS and it is known that high suprathreshold choices can omit components comprising the effect of interest^12^ and low thresholds can yield to non-significant connected components. Here we considered t statistic > 3 as the suprathreshold. The suprathreshold range in which significant group differences were found was 2.8 to 3.6 and we showed the subnetwork corresponding to suprathreshold 2.9. To avoid potential confounding factors, the groups were fully matched for age, sex, handedness and level of education (Table 1).

#### Graph measures

We used a nonparametric test namely random permutation. Permutations (*N* = 100,00) were performed to produce the null distribution of each measure where the p value was calculated and then thresholded using a significance level of *p* < 0.05. The multiple comparison problem was accounted for by controlling the false discovery rate (FDR) at 5%.

## Results

### ROI-wise whole brain functional network comparison

**Fig. 2:**
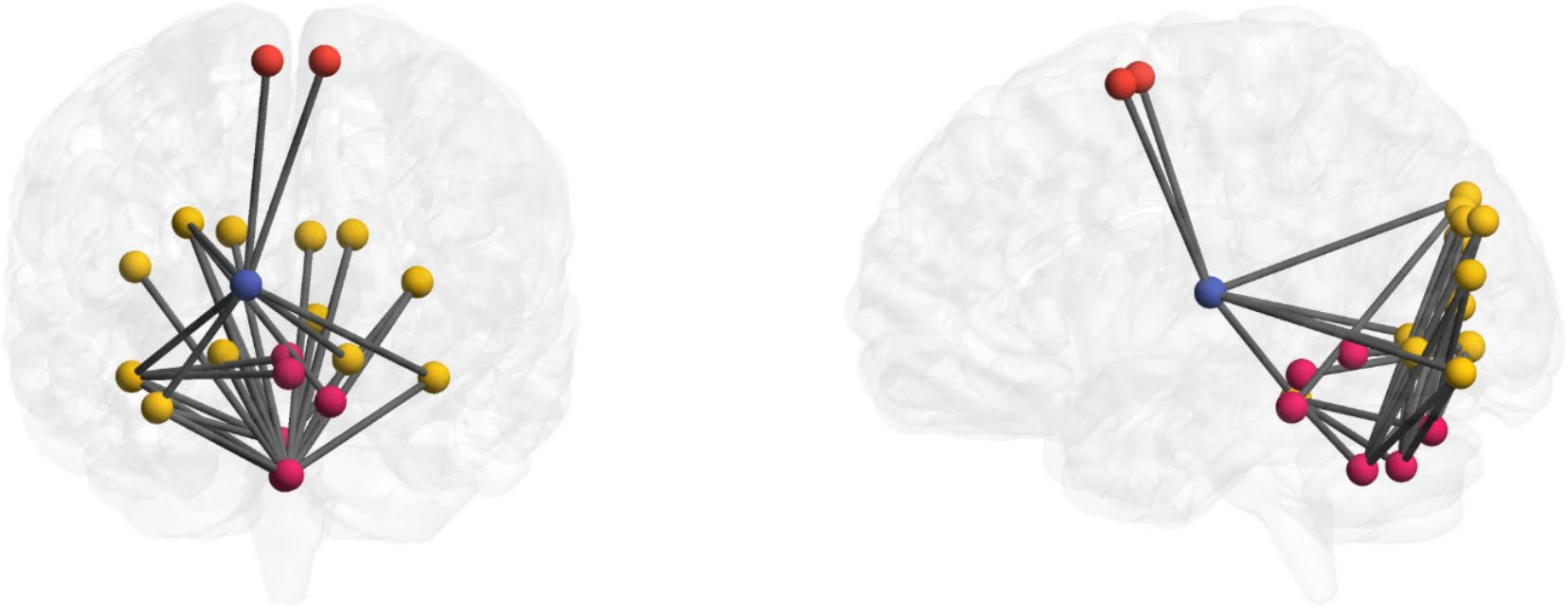
Altered subnetwork in patients with schizophrenia. Patients diagnosed with schizophrenia displayed significantly increased functional resting-state connectivity within a thalamo-cortico-cerebellar network comprising the thalamus (blue), supplementary motor area (red), superior, middle and inferior occipital gyrus (yellow), cuneus, lingual fusiform, calcarine, cerebellum III, vermis VII, VIII, IX (pink). All connections overlaid onto the glass brain were statistically significant between groups (*p* < 0.05 and T > 3) and FWE corrected for multiple comparison. Results visualized using BrainNet viewer^40^.

After statistical analysis using two-sample t-test in NBS (Fig. 1d), we observed a subnetwork (Fig. 2) with significantly increased FC (hyperconnectivity) in the group of schizophrenia patients compared to healthy individuals. The hyperconnected subnetwork comprised thalamo-cortico-cerebellar areas, more specifically the subnetwork consisted of the right thalamus, supplementary motor area, bilateral inferior occipital gyrus, bilateral superior occipital gyrus, right middle occipital gyrus, bilateral cuneus, bilateral lingual, right fusiform, left calcarine, left cerebellum III, vermis VII, VIII, IX (Fig. 2). (*p* < 0.05 and *t* > 3 FWE corrected for multiple comparison).

### Graph analysis

#### Phase1

Group difference across thresholds was found in the degree of thalamus with higher degree in patients with schizophrenia compared with controls (*p* = 0.05 uncorrected).

#### Phase2

No group differences were found after correcting for multiple comparisons.

## Discussion

Despite the fact of a recently growing body of studies analysing FC in schizophrenia, only a few studies directly compared whole brain networks taking all ROIs available^12,15–20,22^ as opposed to selecting a few ROIs a priori. Our exploratory data-driven analysis of whole brain FC revealed increased FC in a thalamico-cortico-cerebellar network in patients with schizophrenia compared to matched healthy individuals. This hyperconnected local subnetwork consisted of the thalamus, supplementary motor area, temporal lobe, occipital lobe, and the cerebellum. Graph analysis showed higher degree in the thalamus. As degree is a measure of centrality in the network that refers to the number of connections of a given node and it is related to the concept of a hub. Therefore, it indicates that the thalamus plays a central role allowing more information to be exchanged across the brain of patients with schizophrenia as compared to controls. Graph analysis at the global scale did not reveal differences between patients with schizophrenia and controls. According to Erdeniz^14^ and Hadley and colleagues^29^ a reason for that might be that clinically stable patients responding to medication regain network connectivity, which therefore might become similar to that of the controls.

Concerning previous studies that used the same methodology as ours, namely NBS to compare whole brain connectivity in a data-driven fashion, patients with schizophrenia showed wide-spread impaired connectivity with heterogeneous multi-site communication in the brain^12,14,16^. However, the same hyperconnected functional subnetwork obtained in the current study was not found in previous studies in patients with chronic schizophrenia using resting-state data in NBS. This is explained by the fact that these studies, excepting one with treatment resistance schizophrenia patients^18^, did not include cerebellar regions as nodes in their whole brain networks. Interestingly, a similar yet much larger hyperconnected thalamo-cortico-cerebellar network was observed in a clinical population with high risk of psychosis when using multi-paradigm fMRI data^19^, however, it was not uncovered using only resting-state whole brain FC data. Also, a multimodal study^15^ showed disruption of structure-function coupling in a subnetwork comprising frontal, temporal, thalamic, and striatal regions in patients with schizophrenia whereas structure-function coupling remained in parietal, occipital, and temporal cortices.

Our finding from exploratory whole brain analysis also aligns with previous studies showing alterations in thalamo-cortico-cerebellar circuits when using different methodology, namely seed-based FC with the seed in the thalamus or ROIs only in the cerebellum: increased connectivity between thalamus and multiple sensory areas, and decreased connectivity between thalamus and the cerebellum^41,42^, cerebellar region showed increased connectivity with the prefrontal cortex and thalamus as well as decreased connectivity with the visual cortex and sensorimotor cortex^43^, disconnections in cognition-related resting state networks^44^, and reduced FC in a thalamo-cortico-cerebellar network^45–47^.

When looking at thalamic and cerebellar functional alterations individually, regarding the thalamus and consistent with our finding is the frequent report of hyperconnectivity between the thalamus and sensorimotor regions^42,48–51^. Importantly, two of these studies performed brain-wide FC analysis on large sample sizes including more than 300 patients each^49,50^. Li et al.^50^ could additionally show that this pattern of thalamic hyperconnectivity is especially pronounced in chronic patients with schizophrenia, which is similar to our sample of chronic patients with a mean illness duration of about 10 years. Regarding the cerebellum, previous studies have also shown altered FC of the cerebellum^43,45,46^. Although the function of the cerebellum is not yet completely understood^52^, multiple models have been proposed in which the role of cerebellum in processing information from the motor cortex is extended to other cortical areas that are involved in higher order cognitive functions^8,53^. Multiple studies have shown structural alterations in this region in patients with schizophrenia including increases in cerebellar volume^54,55^, decreases in cerebellar volume^56–59^ and reductions in grey matter that was associated with thought disorder^60^. Based on the assumed involvement of the cerebellum in a range of cognitive processes, Andreasen and Pierson^61^ hypothesized that pathological alterations within the cerebellum might have the potential to explain heterogeneous symptom characteristics for the diagnosis of schizophrenia.

Bringing the discussion back to our results, the fact that we performed network comparison using all ROIs available in the whole brain network and that we found only an impaired subnetwork and hyper connectivity of the thalamus and no differences in global topological measures, tells us that the disruptions in the brain networks of individuals with schizophrenia are situated only at the local level of the hyperconnected thalamo-cortico-cerebellar subnetwork and not globally spread across the brain. The thalamic-cerebellar interaction forms the basis for cognitive dysmetria theory, since the thalamus is involved in regulating and integrating sensory information^62^ and can be thought as a gateway keeper or a filter. Andreasen^63^ hypothesized that symptoms characteristic for schizophrenia such as hallucinations, delusions, and self-disturbances are due to an overload of information caused by a filtering disruption promoted by the thalamus^4,64^. Moreover, from an information processing perspective, it has been suggested that a disconnection between cerebellum and cortex can lead to a misinterpretation of the information arriving from the cortex, resulting in, for example, experiences of delusion and auditory hallucinations^61^.

Although disruptions in a thalamo-cortico-cerebellar network have been already known from hypothesis-based studies in various populations^41–47,65^. The fact that our local thalamo-cortico-cerebellar subnetwork emerges from a data-driven whole brain analysis emphasizes its importance and robustness. Robustness of results across different studies, methodologies and populations is considered highly important nowadays as fMRI is being criticized by the lack of reliability. Altogether these aspects promote the thalamo-cortico-cerebellar network as a great candidate for a biomarker to be used in translational psychiatry^65,66^. Exploring this direction, in never-medicated patients, similar thalamo-cortico-cerebellar network show a predictive power^65^ and in individuals at ultra-high risk psychosis it predicted positive symptom progression^66^. In addition, hyperconnectivity in a thalamo-cortico-cerebellar subnetwork might be a heritable trait related to the genetic risk of schizophrenia^67^.

However, with the current state of research it is not yet possible to determine whether this hyperconnectivty in thalamo-cortico-cerebellar subnetwork is a cause or rather a consequence of a diagnosis of schizophrenia. To answer this important question, longitudinal studies over a long period of time, preferably containing data before the onset of illness, are needed. Such studies will also be important for the development and effective testing of biomarkers.

### Limitations

Although, our sample size is bigger than previous rs-fMRI studies (35 patients with schizophrenia and 41 healthy individuals) comparing ROI-wise whole brain FC, where all ROIs are taken into account using NBS toolbox in our population with chronic schizophrenia, we acknowledge that the sample size is fairly small. Thus, future research comparing large scale whole brain FC networks using larger sample sizes that also include cerebellar regions are necessary, given the power of data-driven analysis that leads to less bias compared to hypothesis-driven studies where nodes in the thalamo-cortico-cerebellar network are previously selected.

NBS is the best tool to compare large scale ROI-wise brain networks but one downside of this statistical tool is that NBS has power for cluster and not for individual links, for that reason, we cannot make inferences about single links, but only about the subnetwork as a whole which may compromise correlational analysis for large subnetworks.

A further limitation of this study concerns the substance use among the patient cohort. We found however that consumption of alcohol and cannabis was not correlated with the average network connectivity nor connectivity of individual links. Furthermore, the connectivity values of those patients who reported abusing substances were not outliers.

## Conclusion

Together, our results suggest that disruptions in the brain networks of individuals with schizophrenia are situated at the local level of the hyperconnected thalamo-cortico-cerebellar rather than globally spread. The disruption is driven by greater amount of information that is going through the thalamus and reaching sensory and cerebellar areas (and vice-versa) where the thalamus plays a central role in the processing and distributing of this information. Lastly, our results provide further evidence for the importance of the interaction between thalamus and cerebellum and for the notion that the psychopathology of schizophrenia is related to impaired brain networks in line with the dysconnectivity theory and cognitive dysmetria model.

## Electronic supplementary material

Below is the link to the electronic supplementary material.

Supplementary Material 1

## Data Availability

For information about how to obtain the data please contact Prof. Dr. Kühn. The data cannot be stored in public repository as it was not part of the ethics statement. Therefore the participants were not informed that the data would be made public. The codes are freely available upon request.
