# Supplementary material for: Altered resting-state functional connectivity in a thalamo-cortico-cerebellar network in patients with schizophrenia
Source: Sci Rep. 2024 Nov 1;14:26284. doi: 10.1038/s41598-024-78297-3 (PMC11530429; doi:10.1038/s41598-024-78297-3)
Supplement: Supplementary file 1 — Supplementary Material 1 [file 41598_2024_78297_MOESM1_ESM.docx]

**Supplementary material:**

Altered resting-state functional connectivity in a thalamo-cortico-cerebellar network in patients with schizophrenia

Caroline Garcia Forlim ^1,2^, Leonie Klock ^1,3^, Jürgen Gallinat ^1^, Simone Kühn ^1,2*^

^1^Clinic and Policlinic for Psychiatry and Psychotherapy, University Medical Center Hamburg-Eppendorf, Hamburg, Germany

^2^Center for Environmental Neuroscience, Max Planck Institute for Human Development, Berlin, Germany

^3^Berlin School of Mind and Brain, Humboldt-Universität zu Berlin, Berlin, Germany

**Materials and Methods**

**Participants**

Participants were questioned about their substance use with the responses graded along the following scale: “never”, “less than monthly”, “monthly”, “weekly”, “daily”, “abuse” for alcohol and cannabis; “never”, “once”, “past regular use”, “occasionally”, “regularly” for ecstasy, cocaine, hallucinogens, amphetamines, and heroin. Please see Table S1 for a detailed assessment of substance use of both groups. Furthermore, extrapyramidal symptoms of patients diagnosed with schizophrenia were scored with “none”, “light”, “significant”. Three patients showed light extrapyramidal symptoms. Two patients were also taking diazepam as medication on demand.

Table S1 - Self-report substance use

|  | **Frequency** | |
| --- | --- | --- |
|  | **Healthy Participants** | **Schizophrenia Patients** |
| **Alcohol** |  |  |
| never | 12 | 7 |
| less than monthly | 4 | 9 |
| monthly | 1 | 7 |
| weekly | 19 | 6 |
| daily | 4 | 4 |
| abuse | 0 | 1 |
|  |  |  |
| **Cannabis** |  |  |
| never | 37 | 18 |
| less than monthly | 1 | 3 |
| monthly | 2 | 5 |
| weekly | 0 | 1 |
| daily | 0 | 0 |
| abuse | 0 | 7 |
|  |  |  |
| **Ecstasy** |  |  |
| never | 40 | 25 |
| once | 0 | 6 |
| past regular use | 0 | 2 |
| occasionally | 0 | 1 |
| regularly | 0 | 0 |
|  |  |  |
| **Cocaine** |  |  |
| never | 40 | 27 |
| once | 0 | 3 |
| past regular use | 0 | 3 |
| occasionally | 0 | 1 |
| regularly | 0 | 0 |
|  |  |  |
| **Hallucinogen** |  |  |
| never | 38 | 29 |
| once | 0 | 2 |
| past regular use | 0 | 1 |
| occasionally | 0 | 1 |
| regularly | 0 | 0 |
|  |  |  |
| **Heroin** |  |  |
| never | 40 | 32 |
| once | 0 | 0 |
| past regular use | 0 | 1 |
| occasionally | 0 | 1 |
| regularly | 0 | 0 |
|  |  |  |
| **Amphetamine** |  |  |
| never | 40 | 8 |
| once | 0 | 4 |
| past regular use | 0 | 0 |
| occasionally | 0 | 1 |
| regularly | 0 | 2 |
|  |  |  |

**Graph analysis**

The graph measures we used were: degree, which is the total number of connections to an individual node; betweenness that measures the fraction of all shortest paths that pass through an individual node; characteristic path length that measures the average shortest path between all pairs of nodes; efficiency that accounts for the average inverse shortest paths; diameter that is the longest of all shortest paths and cluster coefficient that is the fraction of a node’s neighbours that are also neighbours of each other. For a complete description of the graph measures please refer to [1,2].

**References**

1. Rubinov M, Sporns O. Complex network measures of brain connectivity: Uses and interpretations. Neuroimage. 2010;52: 1059–1069. doi:10.1016/J.NEUROIMAGE.2009.10.003

2. Bullmore E, Sporns O. Complex brain networks: graph theoretical analysis of structural and functional systems. Nat Rev Neurosci. 2009;10: 186–198. doi:10.1038/nrn2575
